# Detailed Analysis of Primary Non-invasive Respiratory Support and Outcomes of Subjects With COVID-19 Acute Hypoxaemic Respiratory Failure

**DOI:** 10.7759/cureus.32362

**Published:** 2022-12-09

**Authors:** James Booker, Rebecca Egglestone, Jack Lushington, Maria Burova, Laura Hamilton, Elsie Hunter, Clare Morden, Darshni Pandya, Ryan Beecham, Robert MacKay, Sanjay Gupta, Michael P Grocott, Ahilanandan Dushianthan

**Affiliations:** 1 General Intensive Care Unit, University Hospital Southampton NHS Foundation Trust, Southampton, GBR; 2 Medicine, University of Southampton, Southampton, GBR; 3 Critical Care, University Hospital Southampton NHS Foundation Trust, Southampton, GBR; 4 Critical Care, University of Southampton, Southampton, GBR

**Keywords:** continous positive airway pressure, pulmonary critical care, acute hypoxemic respiratory failure, non- invasive ventilation, covid 19

## Abstract

Background

The role of non-invasive (continuous positive airway pressure (CPAP) or Non-invasive ventilation (NIV)) respiratory support (NIRS) as a primary oxygenation strategy for COVID-19 patients with acute severe hypoxic respiratory failure (AHRF), as opposed to invasive mechanical ventilation (invasive-MV), is uncertain. While NIRS may prevent complications related to invasive MV, prolonged NIRS and delays in intubation may lead to adverse outcomes. This study was conducted to assess the role of NIRS in COVID-19 hypoxemic respiratory failure and to explore the variables associated with NRIS failure.

Methods

This is a single-center, observational study of two distinct waves of severe COVID-19 patients admitted to the ICU. Patients initially managed with non-invasive respiratory support with laboratory-confirmed SARS-CoV-2 in acute hypoxaemic respiratory failure were included. Demographics, comorbidities, admission laboratory variables, and ICU admission scores were extracted from electronic health records. Univariate and multiple logistic regression was used to identify predictive factors for invasive mechanical ventilation. Kaplan-Meier survival curves were used to summarise survival between the ventilatory and time-to-intubation groups.

Results

There were 291 patients, of which 232 were managed with NIRS as an initial ventilation strategy. There was a high incidence of failure (48.7%). Admission APACHE II score, SOFA score, HACOR score, ROX index, and PaO2/FiO2 were all predictive of NIRS failure. Daily (days 1-4) HACOR scores and ROX index measurements highly predicted NIRS failure. Late NIRS failure (>24 hours) was independently associated with increased mortality (44%).

Conclusion

NIRS is effective as first-line therapy for COVID-19 patients with AHRF. However, failure, particularly delayed failure, is associated with significant mortality. Early prediction of NIRS failure may prevent adverse outcomes.

## Introduction

For the majority, Coronavirus Disease 2019 (COVID-19) is a mild self-limiting illness. However, acute hypoxaemic respiratory failure (AHRF) is a well-recognized feature of severe COVID-19 illness and is associated with significant morbidity and mortality. Studies report that 14-30% of hospitalized subjects may require intensive care unit (ICU) admission for advanced respiratory support [1,2]. For subjects who deteriorate despite supplementary face mask oxygen, invasive or non-invasive mechanical ventilation is often required. Guidelines developed early in the pandemic recommended high-flow nasal oxygen (HFNO) and early intubation rather than non-invasive respiratory support (NIRS), partly due to concerns regarding viral aerosolization and insufficient evidence of its benefit in previous viral outbreaks [3-6]. However, as the pandemic progressed, the use of NIRS increased, partly driven by a lack of intensive care resources [7,8].

Despite this, the best practice approach for NIRS in treating subjects with severe AHRF remains to be determined. Some authors postulate that NIRS in subjects with a high respiratory drive risks patient self-induced lung injury (P-SILI) and propose early intubation [9,10]. While others argue that the evidence for P-SILI is weak and that each day spent on invasive mechanical ventilation (invasive-MV) increases complications and mortality [11,12]. Anecdotal evidence suggests that the prolonged use of NIRS may cause delays in intubation and invasive MV with adverse clinical outcomes [13]. Predicting which patients may fail is often challenging, and achieving this may improve outcomes. Studies in COVID-19 and non-COVID-19 ARDS suggest that NIRS may cause delays in intubation and initiation of invasive MV and may be associated with worse outcomes [14-18].

As part of a locally developed hospital policy, we used NIRS [either CPAP or non-invasive ventilation (NIV)] as a primary ventilation strategy for all new ICU admissions presenting with AHRF secondary to COVID-19 who did not require immediate endotracheal intubation. We have previously presented a preliminary report demonstrating that non-invasive respiratory support is a useful initial oxygenation strategy in patients with moderate to severe COVID-19-associated hypoxaemic respiratory failure and may avoid the need for invasive mechanical ventilation [19]. In this study, we longitudinally explore non-invasive respiratory support (NIRS) and outcomes related to identifiable patient-specific risk factors, several scoring systems, and the duration of non-invasive respiratory support that may help define success or failure.

## Materials and methods

This retrospective observational study was carried out in an adult general ICU in Southampton, UK, with retrospective data collected from 19th March 2020 to 12th March 2021. This was a period that covered two distinct waves of the pandemic in the UK. This study has the ethical approval as part of "a longitudinal cohort study to facilitate better understanding and management of SARS-CoV-2 infection from hospital admission to discharge across all levels of care (REACT-COVID19): REC reference 17/NW/0632, SRB reference number; SRB0025 [20]. Consent was waived due to the retrospective nature of this study. The data collected were anonymized and handled according to the local institutional and national policies. The study used STROBE guidelines for reporting observational studies [21].

We included all patients admitted to our ICU during the study period with laboratory-confirmed SARS-CoV-2 AHRF, requiring fractional inspired oxygen (FiO2) of >60% to maintain an arterial PaO2 of > 60 mmHg, and who were initially managed with NIRS as the primary ventilation strategy. We use the term NIRS to refer to CPAP or NIV but not HFNO. We excluded all subjects transferred in from other hospitals due to challenges in their local bed capacity as we could not obtain their detailed primary ventilation strategy before their transfer. Moreover, subjects managed outside the critical care area, those who received NIRS as the ceiling of ventilatory care, or subjects receiving NIRS as part of weaning from invasive MV were also excluded. The decision as to whether NIRS was the ceiling of care was made by shared decision-making involving more than one consultant physician and the patients and their next of kin, considering the pre-existing comorbidities, functional status, frailty, and patient wishes.

All clinical management was at the discretion of the treating clinical physician and guided by locally produced guidelines. Escalation from NIRS to MV was recommended when FiO2 > 0.6 was required to maintain PaO2 > 60mmHg, respiratory rate > 35, or worsening work of breathing. The need for immediate intubation was defined as invasive MV within the first 2 hours of a NIRS trial. Subjects received NIRS using either a Philips Respironics V60 non-invasive ventilator or a Hamilton-C6 ventilator using the CPAP or NIV mode. Due to the aerosol-generating nature of NIRS and the risk of airborne transmission of the virus, all patients were managed in either negative pressure side rooms or cohorts together, and all healthcare professionals wore appropriate personnel protective equipment while providing care for these patients [22,23]. The interface was via a full facemask or a facemask covering the mouth and nose, depending on the suitable fit and patient comfort. All subjects were encouraged to be self-prone.

Data were extracted from all available electronic health records, including demographics, comorbidities, admission laboratory variables, oxygenation at presentation, admission ICU clinical scores, acute physiology, chronic health evaluation II (APACHE II), and Sequential organ failure assessment (SOFA)), mode and duration of NIRS, need for invasive-MV, and the requirement for other ICU interventions such as renal replacement therapy. HACOR (heart rate, acidosis, consciousness, oxygenation, and respiratory rate) scores and ROX-index were calculated serially from admission every time the observations were documented. From these values, mean scores were obtained for every 24 hours the patient remained on NIRS. The variables for APACHE II, SOFA, HACOR scores, and ROX index are presented in Appendix Table 7 [24-27]. The outcomes reported are the need for invasive-MV, ICU and hospital length of stay, ICU and hospital mortality, and are up to date and complete as of 30/07/2021.

We presented the demographic and outcome categorized into NIRS success and NIRS failure (those who required invasive-MV). We further sub-grouped NIRS failure subjects into three categories depending on the duration (< 24 hours, 24-96 hours, >96 hours) of NIRS before invasive-MV. This study includes data from 79 COVID-19 patients previously presented as preliminary dissemination of the intervention during the rapidly evolving 1st COVID-19 wave in the UK [19].

Continuous variables are reported as the median and interquartile range (IQR), and categorical variables are reported as numbers and percentages. Variables that differed between NIRS success and NIRS failure groups were inspected. Continuous variables with non-normal distributions were transformed into dichotomized variables according to recognized cut-off values. Univariate logistic regression was used to identify predictive factors for invasive MV, and a post-hoc Bonferroni correction was done to account for multiple testing. Factors with a p-value <0.5 in the univariate analysis were considered statistically significant. A correlation analysis was done to assess multiple collinearities between significant predictors. This informed the inclusion of variables in the multivariate logistic regression model. The accuracy of variables in predicting the need for intubation was assessed using the area under receiver operating curves (AUROC) with optimal cut-off calculated using Youden's index. Kaplan-Meier survival curves have been used to summarise survival between the different ventilatory and time-to-intubation groups. Cox-proportional hazard analysis, adjusted for age, ethnicity, and Charlson comorbidity index (CCI), was conducted to assess their effect on hospital mortality. Statistical analysis was performed using SPSS version 27, R version 1.2.5042, and MedCalc version 20.008.

## Results

**Figure 1:**
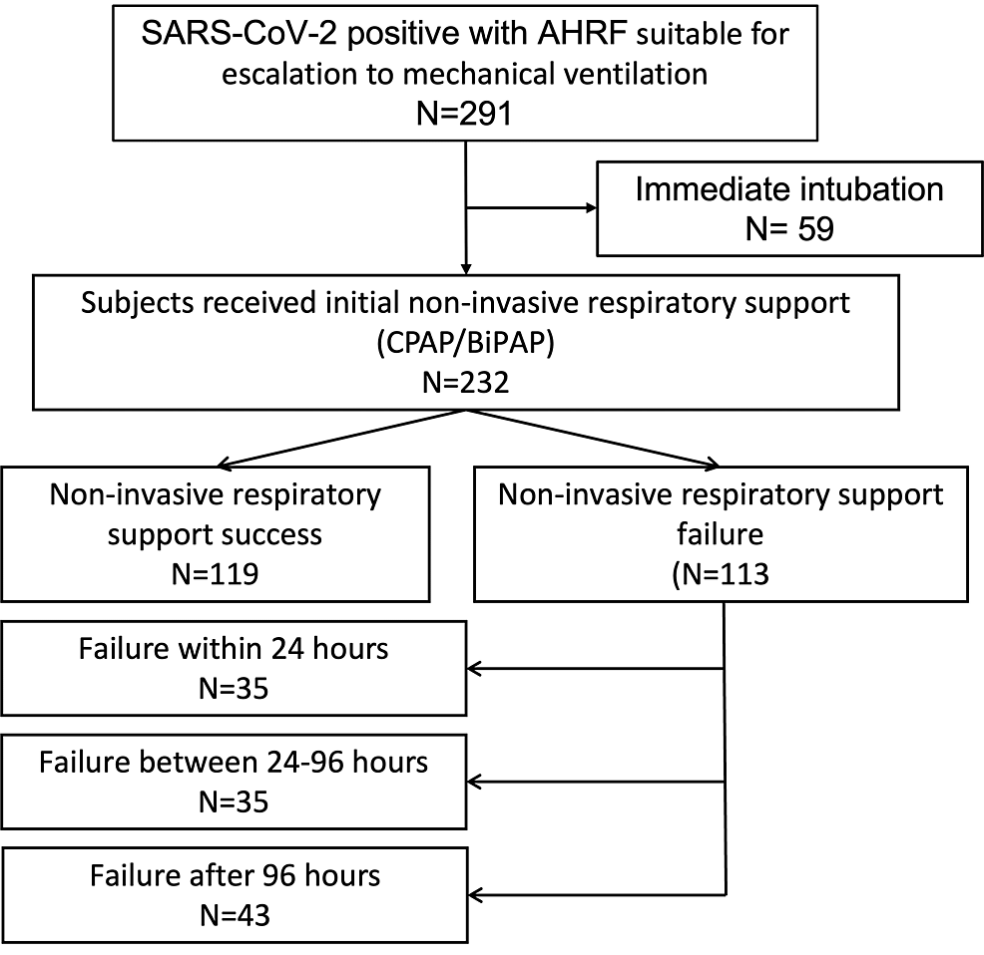
Initial ventilatory management of SARS-CoV-2 positive subjects admitted to the intensive care unit. CPAP- Continuous positive airway pressure; AHRF- Acute severe hypoxic respiratory failure; BiPAP- Bilevel Positive Airway Pressure

Between 03/2020 and 03/2021, 291 SARS-CoV-2 confirmed subjects were admitted with severe AHRF suitable for escalation to invasive MV. Of these, 232 (79.7%) were initially managed with NIRS, and the remaining 59 (20.3%) required immediate intubation and invasive mechanical ventilation. We subdivided the NIRS trial group (CPAP/NIV) into two groups, NIRS successful and NIRS failure. Of those with NIRS use, 119 (51.3%) subjects were successfully managed, and 113 (48.7%) failed with the following requirements for intubation and invasive MV (Figure 1). Table 1 summarises the baseline characteristics of all NIRS patients.

**Table 1:** Characteristics of subjects with severe COVID-19 treated with non-invasive respiratory support. Each parameter is expressed as median (interquartile range) or number (percentage). BMI: body mass index; eGFR: estimated glomerular filtration rate; EPAP: expiratory positive airway pressure; IPAP: inspiratory positive airway pressure; INR: international normalised ratio; N/L ratio: neutrophil/lymphocyte ratio; NIV: non-invasive ventilation; SOFA: sequential organ failure assessment score; WCC: white cell count

	All subjects N= 232	Non-invasive respiratory support Success N= 119	Non-invasive respiratory support Failure † N= 113
Baseline characteristics			
Age (years)	58 (49 – 67)	56 (45 – 66.5)	59 (52 – 67)
Sex, male (n, %)	148 (63.8%)	79 (66.4%)	69 (61.1%)
BMI (kg/m^2^)	31.2 (27.0-36.3)	32.1 (27.2 – 36.2)	31.0 (26.9 – 36.3)
Ethnicity, white (n, %)	180 (77.6%)	100 (84.0%)	80 (70.8%)
Clinical Frailty Score	2 (2-3)	2 (2-3)	2 (2-3)
Charlson Comorbidity index	2 (1-3.3)	1 (0-3)	2 (1-4)
Admission ICU Scores and oxygenation			
APACHE II score	12 (9-16)	10 (8-13)	13 (10-20)
SOFA score	4 (3-4)	3 (3-4)	4 (3-5)
ROX index	5 (4-7)	6.0 (5-8)	5 (3-6)
HACOR score	5 (4-6)	5.0 (3-6)	6.0 (5-7)
PaO_2_/FiO_2_ (kPa) ratio	13.5 (10.5-16.4)	14.7 (12.9 – 18.2)	11.7 (9.0-14.2)
Admission laboratory variables			
Bilirubin (µmol/L)	10 (8-13)	10 (8-12)	11 (8-13)
Urea (mmol/L)	6.6 (5.0-9.2)	6.5 (4.8-9.1)	6.8 (5.4-9.4)
Creatinine (mmol/L)	69 (56-92)	67 (57-91)	70 (56-96)
eGFR (ml/min/1.73m^2^)	90 (71-90)	90 (77 – 90)	90 (70-90)
WCC (10^9^/L)	8.3 (5.8 -11.1)	8.1 (5.7-11.1)	8.4 (6 -11.1)
Neutrophils (10^9^/L)	6.8 (5.0-9.7)	6.5 (4.8-9.5)	7.5 (5.1-9.7)
Lymphocytes (10^9^/L)	0.8 (0.6-1.1)	0.8 (0.6-1.2)	0.7 (0.5-0.9)
N/L ratio	8.6 (5.8-14.0)	7.3 (5.2-11.5)	9.3 (6.6-16.9)
C- reactive protein (mg/L)	128 (78-195)	125 (73-190)	134 (81-201)
INR	1.1 (1.0-1.2)	1.1 (1.0-1.2)	1.1 (1.1-1.2)
Ferritin (mg/L)	742 (398-1370)	685 (406-1446)	776 (394-1261)
Troponin (ng/ml)	11 (6.8-23.5)	9 (6-22)	13 (8-29)
D-dimer (ng/ml)	486 (272-952)	400 (233-896)	578 (297-1096)
Procalcitonin (ng/ml)	0.2 (0.1-0.7)	0.2 (0.1-0.4)	0.3 (0.2- 0.9)
HbA1c (mmol/mol)	46 (41-56)	44 (40-55)	48 (42-59)
Lactate dehydrogenase (U/L)	925 (757-1235)	866 (694-1228)	1000 (816-1250)
Non-invasive respiratory support parameters			
Duration, hrs	88.0 (35.8-144.0)	99.0 (72.0-189.0)	48.0 (17.0-114.0)
Mode: NIV only, CPAP only, and Both	34 (14.7%), 107 (46.1%), 91 (39.2%)	13 (10.9%), 66 (55.5%), 40 (33.6%)	21 (18.6%), 41 (36.3%), 51 (45.1%)
NIV pressures (cmH_2_O) IPAP EPAP	16 (14-18) 10 (10-12)	16 (14-18) 10 (8-12)	16 (12-18) 10 (10-12)
CPAP pressure (cmH_2_O)	10 (8-12)	10 (8-10)	10 (9-12)

Gender, body mass index (BMI), and clinical frailty scores were similar between groups. Group differences were noted in variables of age, ethnicity, CCI, admission APACHE II score, SOFA score, PaO_2_/FiO_2_ (P/F) ratio, HACOR score, ROX index, and several admission laboratory variables, including lymphocytes, neutrophil/lymphocyte (N/L) ratio, troponin, d-Dimer, procalcitonin (PCT), HbA1C and lactate dehydrogenase. Subjects with NIRS success were also more likely to be managed with CPAP only (55.5% vs. 36.3%) than with a combination of CPAP and bilevel non-invasive ventilation (Table 1).

The variables that differed between the group were inspected. Variables were dichotomized if they were not normally distributed according to widely-accepted cut-off values. We then performed a univariate logistic regression analysis for the outcome of NIRS failure with progression to invasive mechanical ventilation. We corrected for multiple testing with a Bonferroni correction. This demonstrated that admission APACHE II score, SOFA score, ROX index, HACOR score, PaO2/FiO2 ratio < 13.3 kPa (<100mmHg), and N/L ratio all influenced progression to intubation (Table 2). We then assessed the multicollinearity between the significant variables with a correlation matrix using spearman's rank method. This showed that admission APACHE II, HACOR, and N/L ratio were not significantly correlated (see Appendices Figure 6). These variables were selected in a multivariate logistic regression predicting the outcome of NIRS failure with progression to invasive mechanical ventilation (Table 2).

**Table 2:** Univariate and multivariate logistic regression analysis for variables that varied between non-invasive respiratory support success and failure groups. P value adjusted with Bonferroni correction. *p<0.05 significant. APACHE II: acute physiology and chronic health evaluation II; CPAP: continuous positive airway pressure; EPAP: expiratory positive airway pressure; HACOR: heart rate, acidosis, consciousness, oxygenation, respiratory rate score; N/L ratio: neutrophil/lymphocyte ratio; NIV: non-invasive ventilation; PCT: procalcitonin; ROX index: respiratory rate-oxygenation index; SOFA: sequential organ failure assessment score.

Univariate analysis				
Variable	Odds ratio		95% CI	Adjusted p-value
Baseline characteristics				
Age, years	1.02		1.00-1.04	0.73
Ethnicity, white	0.46		0.24-0.87	0.46
Charlson comorbidity index	1.16		1.01-1.35	1.00
Admission ICU scores and oxygenation				
APACHE II score	1.19		1.12-1.27	< 0.001*
SOFA score	1.71		1.38-2.20	< 0.001*
ROX Index	0.74		0.64-0.84	< 0.001*
HACOR score	1.40		1.22-1.62	< 0.001*
PaO_2_/FiO_2_ ratio < 13.3 kPa	2.71		1.48-5.11	0.04*
Admission laboratory variables				
Lymphocytes low (<1.0 x 10^9^/L)	2.54		1.39-4.75	0.08
N/L ratio	1.06		1.03-1.10	0.03*
Troponin high (>30 ng/ml)	1.28		0.68-2.40	0.19
D-Dimer > 500 (ng/ml)	2.16		1.27-3.72	0.14
Procalcitonin high (≥0.3 ng/ml)	2.23		1.25-4.03	0.19
HbA1c > 48 (mmol/mol) (n=156)	2.31		1.22, 4.45	0.30
Lactate dehydrogenase high (>280 U/L)	1.00		1.00-1.00	1.00
Non-invasive respiratory support parameters				
Duration, hrs		0.99	0.99-1.00	1.00
Mode CPAP only		0.54	0.25-1.1	1.00
NIV pressures (cmH_2_0) EPAP		1.26	1.07-1.50	0.21
CPAP pressure (cmH_2_0)		1.15	1.00-1.32	1.00
Multivariate analysis				
APACHE II score		1.18	1.11-1.27	<0.001*
HACOR score		1.35	1.17-1.59	<0.001*
N/L ratio		1.05	1.02-1.10	0.01*

The median CPAP pressure was 10cmH_2_O, and IPAP of 16cmH_2_O with an EPAP of 10cmH_2_O was used as initial NIV settings. The use of CPAP alone was proportionately higher in the NIRS success group. For all subjects who received NIRS, the median duration of support was 88 hours (IQR 35.8-144.0 hours). Among those who failed non-invasive respiratory support, the median duration before needing invasive mechanical ventilation was 48 hours (IQR 17.0-114.0). Subjects successfully managed with NIRS needed a median duration of therapy of 99 hours (IQR 72.0-189.0).

We further sub-categorized the NIRS failure group depending on the duration of therapy before needing invasive MV. For patients initially managed with NIRS and then went on to require intubation, 35 (31.0%) were intubated within 24 hours, 35 (31.0%) within 24-96 hours, and 43 (38%) after more than 96 hours. Appendix table 6 shows the differential demographics of these patients. There were significantly more males in the prolonged non-invasive respiratory support group (>96 hours) compared with the group who had < 24 hours of non-invasive respiratory support. Those intubated within 24 hours had significantly higher APACHE II, SOFA scores, CRP, and INR on admission, suggesting that this group was more clinically unwell on admission to critical care.

**Figure 2:**
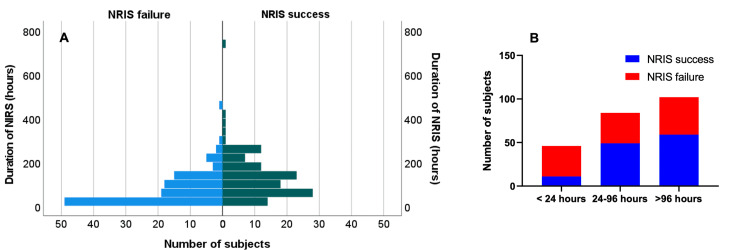
Pictural representation of the duration of non-invasive ventilation in all NIV patients stratified according to if they required IMV after the NIV trial (A). Subcategories according to the duration of NIV with group proportions (B). IMV: invasive mechanical ventilation.

The total duration of non-invasive respiratory support in both groups (non-invasive respiratory support success and failure) are presented pictorially in Figure 2A. For all patients who had non-invasive respiratory support (N=232), 19.8% (N=46) had it for <24 hours, 36.2% (N=84) for 24-96 hours, and 44.0% (N=102) for >96 hours. The NIRS failure rate was proportionately less, with increased duration at 76.1%, 41.7%, and 42.2% for durations <24 hours, between 24-96 hours, and >96 hours of support, respectively (Figure 2B). This suggests that the longer the non-invasive respiratory support duration, the less likely the patients will fail.

The overall hospital survival for all patients admitted to critical care for a trial of NIRS was 82.8% (N=192). All NIRS patients, appropriate for invasive MV but did not require eventual mechanical ventilation, survived hospital discharge. By comparison, the hospital survival for those who required invasive MV after a trial of NIRS was 64.6%. However, there were variations in hospital mortality depending on the duration of NIRS offered before invasive MV. The hospital survival for groups with NIRS duration of <24 hours, 24-96 hours, and >96 hours were 82.9%, 60.0%, and 53.5%, respectively (Table 3). There were no differences in the duration of mechanical ventilation, ICU, or hospital length of stay between these three groups according to the NIRS duration.

**Table 3:** Outcome differences in subjects who failed non-invasive respiratory support and required invasive mechanical ventilation. Expressed as median (interquartile range) or number (percentage). Continuous parameters were compared using the Kruskal-Wallis test, and proportions were compared using Fisher’s exact test.  *Indicates significant (p<0.05) group differences. HLOS: hospital length of stay; ICULOS: intensive care unit length of stay; MV: mechanical ventilation; NIRS: non-invasive respiratory support.

	All subjects n = 113	NIRS < 24 hours n = 35	NIRS 24-96 hrs n = 35	NIRS >96 hrs N= 43	p-value
Duration of MV (days)	15.0 (9.8-25.0)	16.0 (13.0-25.0)	15.0 (10.0-28.0)	13.0 (8.5-23.5)	0.5639
ICULOS (days)	22.0 (15.0-38.0)	21.0 (15.5-35.0)	21.0 (12.0-37.0)	23.0 (17.0-45.5)	0.4410
HLOS (days)	31.0 (21.5-55.0)	30.0 (21.5-51.0)	28.0 (21.3-46.3)	38.5 (23.3-66.3)	0.2275
ICU survival	76 (67.3%)	29 (82.9%)	22 (62.9%)	25 (58.1%)	0.0551
Hospital survival	73 (64.6%)	29 (82.9%)	21 (60.0%)	23 (53.5%)	0.0208*

**Figure 3:**
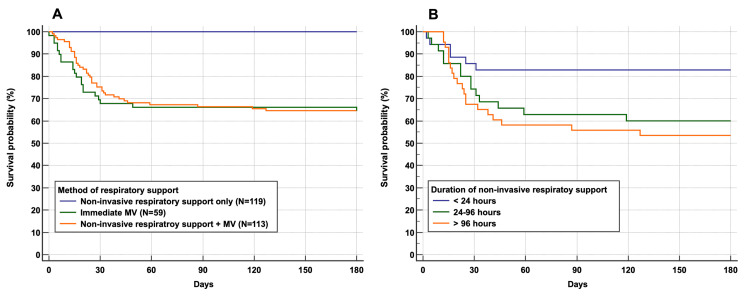
Kaplan-Meir survival curves according to A) Mode of ventilation and B) NIV failure subjects stratified according to the duration of non-invasive ventilation before intubation.

**Figure 4:**
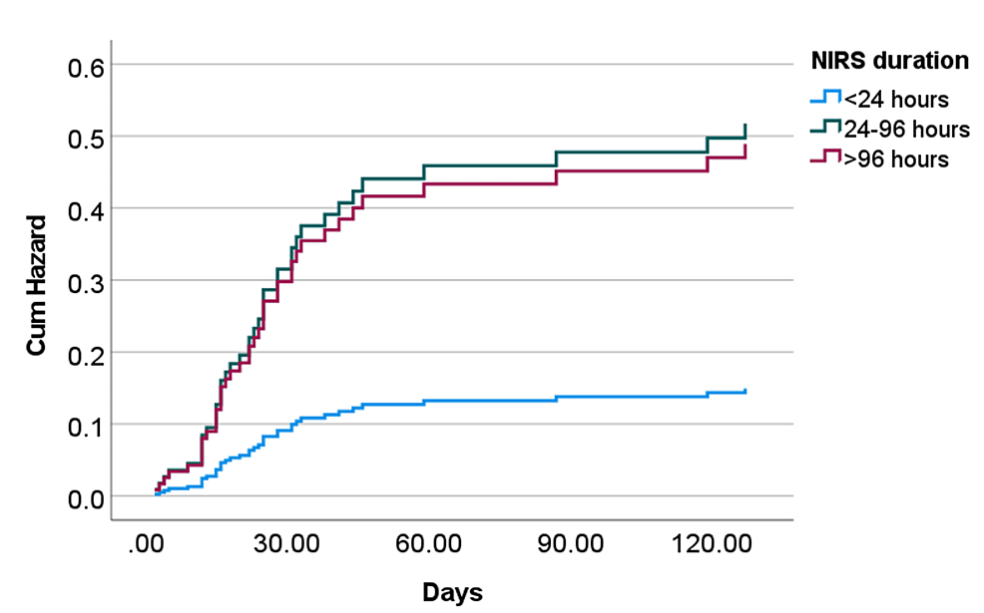
Age, ethnicity, and Carlson Comorbidity Index adjusted Cox proportional hazard for overall hospital mortality according to the duration of NIV before intubation in subjects who failed NIV. NIV- Non-invasive ventilation

Kaplan-Meir survival curves demonstrate significant survival differences between the NIRS success group and who received mechanical ventilation either immediately or following NIRS failure (Figure 3A). Moreover, there were significant differences in mortality in the NIRS failure group according to the duration of NIRS before invasive mechanical ventilation (Figure 3B). For the NIRS failure group, Cox- proportional analysis adjusted for age, ethnicity, Charlson comorbidity index, and APACHE II score suggests an increased risk of hospital death in those intubated between 24-96 hours (Odds ratio 3.46 (95% CI 1.25-9.58, p=0.0168) and after 96 hours (Odds ratio 3.27 (95% CI 1.42-8.74, p=0.0181) compared to those intubated within 24 hours of NRIS initiation (Figure 4).

**Figure 5:**
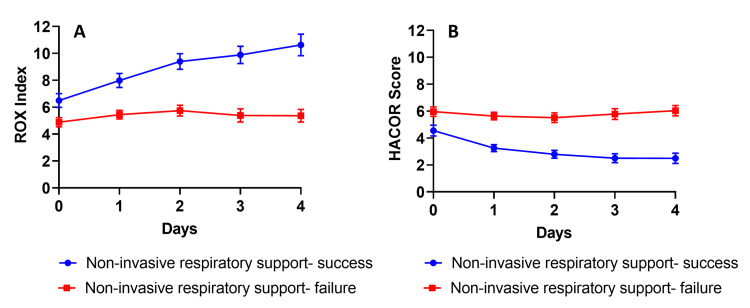
The longitudinal dynamic changes in ROX index and HACOR scores over the first four days of ICU admission for both success and failure groups. *P<0.05.

**Figure 6:**
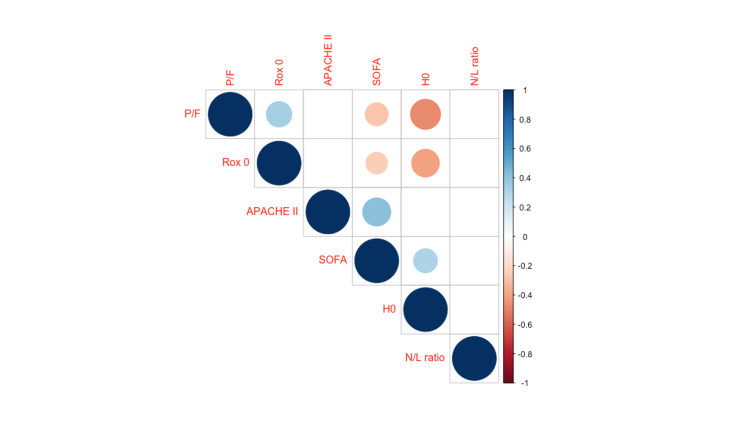
Correlation matrix of significant predictors of non-invasive ventilation failure and subsequent intubation. Correlation matrix of significant predictors of a univariate analysis predicting non-invasive ventilation failure. Correlation calculated using Spearman's rank method with significant correlations defined as p<0.01. Blue circle signifies positive correlation and red circle a negative correlation. APACHE II: acute physiology and chronic health evaluation II; H0; admission heart rate, acidosis, consciousness, oxygenation, respiratory rate (HARCOR) score; N/L ratio: neutrophil/lymphocyte ratio; ROX: respiratory rate-oxygenation index; SOFA: sequential organ failure assessment score.

To predict NIRS failure, we performed longitudinal ROX-index and HACOR scores for all patients up to 4 days from ICU admission (Figure 5). There was a clear separation of both the ROX index and HACOR scores between both groups. In the success group, both scores improved over time. In comparison, the NIRS failure group had no increment in the ROX index and remained at a score of 5, even on day 4. Similar findings were also seen with the HACOR scores, where the NIV success group had a continuous decline in HACOR scores over the four days.

The multivariate logistic regression adjusted for age, ethnicity, and comorbidities (CCI), suggests a significantly increased risk of intubation when these variables are dichotomized (as > 5 for HACOR score and < 5 for ROX index- median admission values) if NIRS did not improve the ROX index and HACOR scores from admission values (Table 4). The ROC analysis revealed that both scores highly predict NIV failure as the day progresses (Table 5). The most predictive variable of intubation was the HACOR score at day 4 (AUC 0.942, 95% CI 0.903-0.981, p<0.001), followed by the ROX index at day 4 (AUC 0.924, 95% CI 0.880-0.968, p<0.001) (Table 5).

**Table 4:** Multivariate logistic regression for ROX index and HACOR scores (day 1-4 ICU admission), adjusted for age, ethnicity and Charlson comorbidity index (CCI) for predicting non-invasive respiratory support failure and intubation. *p<0.05 significant. HACOR: heart rate, acidosis, consciousness, oxygenation, respiratory rate score; N/L ratio: neutrophil/lymphocyte ratio; ROX index: respiratory rate-oxygenation index.

Variable	Logistic regression		
	Odds ratio	95% CI	p-value
Day 1			
ROX index (N=231)	0.54	0.45-0.66	<0.001
ROX index <5	7.32	3.86-13.86	<0.001
HACOR score (N=233)	2.61	2.06-3.34	<0.001
HACOR score >5	11.88	5.89-23.99	<0.001
Day 2			
ROX index (N=181)	0.48	0.38-0.60	<0.001
ROX index <5	11.16	4.67-26.65	<0.001
HACOR Score (N=182)	2.90	2.11-3.98	<0.001
HACOR score >5	14.24	5.69-35.59	<0.001
Day 3			
ROX index (N=148)	0.42	0.31-0.56	<0.001
ROX index <5	44.37	13.33-147.73	<0.001
HACOR Score (N=146)	3.37	2.23-5.10	<0.001
HACOR score >5	36.94	11.52-118.41	<0.001
Day 4			
ROX index (N=120)	0.36	0.23-0.56	<0.001
ROX index <5	13.87	4.59-41.93	<0.001
HACOR Score (N=119)	5.85	2.62-13.06	<0.001
HACOR score >5	49.44	12.86-190.11	<0.001

**Table 5:** The Receiver Operating Characteristics for ROX index and HACOR scores (Day 1-4 in ICU) in predicting non-invasive respiratory support failure. p<0.05 significant. HACOR: heart rate, acidosis, consciousness, oxygenation, respiratory rate score; N/L ratio: neutrophil/lymphocyte ratio; ROX index: respiratory rate-oxygenation index.

	AUC	95% CI	Threshold	Sensitivity	Specificity	p-value
Day 1						
ROX index	0.788	0.732-0.845	≤6	77.68	66.39	<0.001
HACOR score	0.854	0.806-0.902	>4	77.68	81.51	<0.001
Day 2						
ROX index	0.865	0.814-0.916	≤6	74.29	84.68	<0.001
HACOR score	0.884	0.837-0.931	>4	76.47	85.96	<0.001
Day 3						
ROX index	0.906	0.860-0.952	≤7	86.27	76.29	<0.001
HACOR score	0.916	0.874-0.958	>3	95.65	72.00	<0.001
Day 4						
ROX index	0.924	0.880-0.968	≤6	84.85	86.21	<0.001
HACOR score	0.942	0.903-0.981	>4	96.87	82.76	<0.001

## Discussion

This single-center retrospective observational study evaluated the outcomes of patients with AHRF secondary to COVID-19 and managed with NIRS as the primary oxygenation strategy. Around half of the patients trialed with NIRS for a minimum of 2 hours failed this trial and required escalation to invasive MV. We also sought to determine the impact of delayed intubation on outcomes. We discovered a significantly greater mortality rate in those receiving > 96 hours of NIRS before intubation versus those receiving < 24 hours (46.5% vs. 17.1%). From our data, we were also able to determine that several variables were predictive of NIRS failure, including APACHE II score (OR 1.19; 95% confidence interval (CI) 1.12-1.27), PaO2/FiO2 ratio < 13.3 kPa (OR 2.71; 95% CI 1.48-5.11) and HACOR score (OR 1.4; 95% CI 1.22-1.62). Furthermore, the HACOR score and ROX-index divergence between NIRS success and failure groups were significant from day one through day four.

Our failure rate of NIRS (49%) in COVID-19 patients was consistent with previously published literature [28-30]. We adjusted for multiple testing and identified a higher APACHE II score, SOFA score, and HACOR score, a lower ROX-index, and a PaO2/FiO2 ratio < 13.3 kPa and N/L ratio were all independent predictors for NIRS failure. Similarly, a study from China also found age, comorbidities, and ROX-index predictive of NIRS failure in COVID-19 [31]. In addition, this study also found GCS and the use of vasopressors to be predictive. While we did not report on this, they are reflected in the APACHE II, SOFA, and HACOR scores. Other studies report increased minute ventilation on day one, raised C-reactive protein (CRP|), and elevated D-Dimer levels associated with NIRS failure [18,32]; contrary to our data CRP did not differ between the NIRS success and failure groups.

We further subdivided the NIRS failure subjects depending on the duration of NIRS before the initiation of IMV. Among those who failed, 31% required invasive MV within 24 hours, 31% between 24-96 hours, and 38% beyond 96 hours after the NIRS initiation. The subjects intubated within 24 hours had a median NIRS duration of 9 hours and, unsurprisingly, had significantly higher APACHE II, SOFA, and HACOR scores on admission. Mortality rates were found to be increased with increased duration of NIRS before intubation (17.1% in those intubated within 24 hours vs. 46.5% in those intubated after 96 hours), despite those being intubated within 24 hours had higher illness severity scores presentation. In comparison, the overall hospital survival for all patients in this study was 79%. Hospital survival for those eligible for mechanical ventilation but who had a successful trial of NIRS was 100%, decreasing to 64.6% for those failing their trial.

While these results imply that delayed IMV and prolonged NIRS trials may worsen clinical outcomes, the clinical interpretation is likely to be complex and multifactorial. Few studies report on the duration of NIRS and outcome in COVID-19, and none, to the best of our knowledge, are specific to critical care. Boscolo et al. found that using NRIS more than 48 hours before intubation in environments outside critical care was an independent risk factor for inpatient mortality [17]. Vaschetto et al. also demonstrated higher in-hospital mortality for those managed with CPAP for more than three days before intubation, where CPAP was delivered outside critical care [18]. It is unclear whether there is a causal relationship between NIRS duration and mortality, and further randomized clinical trials will be required to explore this concept. However, given the increased mortality and morbidity associated with intubation, a balance must be maintained between early intubation for those most likely to fail and avoid it for those who will not benefit from it. In the intensive care setting, nearly half of the COVID-19 subjects trialed with NIRS will go on to need endotracheal intubation. However, a successful NIRS trial is associated with a significantly better outcome than those who receive invasive MV. Early prediction of those most likely to fail may help minimize intubation delay.

There was a clear difference in the trend in HACOR and ROX index scores from admission onwards, and it can help predict those at risk. The ROX index and HACOR scores are equally highly predictive of NIRS failure as the duration of its use increases. Similar to our data, Liu et al. demonstrated that the ROX index was an independent risk factor for failure of NIRS in patients with COVID-19 [31]. In non-COVID-19 patients with AHRF, a ROX index of <4.88 and a HACOR score > 5 are predictive of HFNO and NIV failure, respectively [26,27]. The simplistic nature of the ROX index may enable rapid identification of patients at risk of NRIS failure outside the ICU without additional invasive monitoring. When there is a severe hypoxic respiratory failure with a PaO2/FiO2 ratio of <13.3 kPa on admission with persistently high HACOR scores (>5) and low ROX index (<5), these patients appear to be at risk of needing invasive-MV and should warrant early assessment for its consideration. When NIRS is applied, over the time course, there is a divergent response, where some had no changes from their admission HACOR score/ROX index values with an eventual progression to invasive-MV, and prompt recognition of such subjects in advance may prevent delayed intubation.

During the early course of the pandemic, initial uncertainty regarding the efficacy of NIRS in COVID-19 AHRF coupled with the potential risk of aerosolization, resulted in increased skepticism towards using NIRS in this group. Consequently, guidelines during the pandemic's initial stages preferred HFNO to NIV/CPAP [5]. However, from the pandemic's start, we developed local guidance for using NIRS as either CPAP or NIV as a primary ventilation strategy, with HFNO only used during NIV break periods. However, as the pandemic progressed, randomized controlled trials showed CPAP, compared to HFNO, to be associated with reduced rates of invasive MV [33,34], so we continued our practice in line with this evidence.

Our data suggest that delayed NIRS failure in some patients may be associated with worse outcomes in COVID-19-related severe AHRF. As this was a retrospective observational study, we cannot determine whether this finding is due to the progression of the disease process, regardless of any interventions provided, or if this is genuinely related to delayed invasive MV. Both immediate intubation and late NRIS failure were associated with increased mortality. This is likely a reflection of the severity of the illness than the duration of NIV or other measures instituted. Moreover, those intubated late may have worse outcomes due to the possibility of P-SILI worsening the underlying lung pathology or the presence of different clinical phenotypes of COVID-19. Data from an Italian series found that those with higher D-Dimers, as a surrogate for thromboembolic disease, and lower lung compliance had worse 28-day mortality than those with lower D-Dimers and better lung compliance [35]. In our cohort, the admission D -Dimer was higher in those that failed NIRS but did not differ between the three groups according to the duration of NIRS before failure. Gattinoni and colleagues proposed two separate phenotypes, an L-type characterized by low elastance and low lung recruitability and an H-type characterized by high elastance [36]. The presence of differing phenotypes may partly explain why those intubated early seem to do better than those intubated late. Further studies are needed to help clarify this.

Our study has several limitations. This was a retrospective, observational, single-center study, and clinical practices may not be reflective or generalizable across different centers. Consecutive subjects were enrolled in the study to minimize selection bias. Furthermore, we only collected some potential variables, such as invasive-MV ventilation variables post-intubation, which may have contributed to the patient's outcomes. Moreover, except for the ROX index and HACOR score, we did not collect dynamic clinical variables throughout the disease process, which could provide additional details on disease progression. Nevertheless, we have shown that nearly 50% of subjects with AHRF associated with COVID-19 may improve without requiring invasive MV with significantly better outcomes than those requiring invasive MV. While awaiting further clarification from clinical trials comparing invasive-MV vs. NIRS, NIRS does have a definite role in moderate to severe COVID-19-related AHRF.

## Conclusions

This retrospective observational study shows that subjects with AHRF, secondary to COVID-19, managed solely with NIRS have better outcomes than those intubated immediately or who go on to require invasive mechanical ventilation after NIRS, with PaO2/FiO2 ratio, APACHE II score, ROX index, and HACOR scores appear to predict the need for intubation. Delayed NIRS failure (>24 hours) may be associated with increased hospital mortality; the reasons for this remain unclear and warrant further studies. The daily trend in HACOR scores and ROX index appear reliable in predicting NIRS failure and may allow for early intubation in those most likely to fail.

## Appendices

**Table 6:** Patient characteristics of subjects requiring intubation according to duration of preceding non-invasive respiratory support. Each parameter is expressed as median (interquartile range) or number (percentage). Comparisons carried out using the Kruskal-Wallis test for continuous variables and Fisher’s exact test for categorical variables. *Indicates significant p<0.05 group differences. APACHE II: acute physiology and chronic health evaluation II; BMI: body mass index; eGFR: estimated glomerular filtration rate; EPAP: expiratory positive airway pressure; HACOR: heart rate, acidosis, consciousness, oxygenation, respiratory rate score; IPAP: inspiratory positive airway pressure; INR: international normalised ratio; N/L ratio: neutrophil/lymphocyte ratio; NIV: non-invasive ventilation; PCT: procalcitonin; ROX index: respiratory rate-oxygenation index; SOFA: sequential organ failure assessment score; WCC: white cell count.

Variables	NIRS Failure within 24 hours N=35	NIRS Failure at 24 - 96 hours N=35	NIRS Failure after 96 hours N=43	p-value
Baseline characteristics				
Age, years	59 (50-70)	58 (52-64)	60 (55-68)	0.393
Sex, male	19 (54.0%)	17 (48.6%)	33 (76.7%)	0.025*
BMI (kg/m^2^)	29.6 (25.6-37.3)	33.7 (29.0-37.0)	30.0 (27.2-35.0)	0.312
Race, white	21 (60.0%)	29 (82.9%)	30 (69.8%)	0.108
Clinical frailty score	2 (1-3)	2.5 (2-4)	2 (2-3)	0.350
Charlson comorbidity index	2 (0.5-4)	3 (1.0-3.5)	3 (2-3.5)	0.456
Admission ICU scores and oxygenation				
APACHE II score	19.0 (12-26)	13 (11-17)	13 (10-17)	0.008*
SOFA score	5 (3.5-6.5)	4 (3.5-5.0)	3 (3.0-4.0)	0.003*
ROX index	5 (4-6)	4 (3-6)	5 (4-6)	0.527
HACOR score	5 (4-6)	6 (5-6)	6 (5-7)	0.028*
PaO_2_/FiO_2_ ratio (kPa)	11.0 (8.5-14.4)	11.7 (9.0-13.6)	11.7 (9.7-14.5)	0.613
Admission laboratory variables				
Bilirubin (µmol/L)	12 (8-16)	11 (8-13)	10 (7-13)	0.608
Urea (mmol/l)	6.2 (4.5-9.6)	6.7 (5.6-9.6)	7.0 (6.0-9.3)	0.311
Creatinine (mmol/l)	83 (50-98)	69 (56 - 115)	70 (62-83)	0.980
eGFR (ml/min/1.73m^2^)	87 (66-90)	90 (55-90)	90 (80-90)	0.751
WCC 10^9^/l	9.0 (6.8-11.6)	7.9 (5.5-10.2)	8.4 (6.7-11.2)	0.234
Neutrophils (10^9^/L)	7.7 (5.1-10.7)	6.5 (4.9-9.0)	7.2 (5.7-10.0)	0.389
Lymphocytes 10^9^/l	0.8 (0.5-1.0)	0.6 (0.5-0.8)	0.7 (0.5-0.9)	0.481
N/L ratio	10.7 (5.5-16.5)	8.8 (6.9-16.9)	9.3 (7.1-16.3)	0.989
C-reactive protein (mg/L)	165 (119-246)	123 (79 - 177)	102 (66-160)	0.004*
INR	1.2 (1.1-1.3)	1.1 (1.1-1.2)	1.1 (1.1-1.2)	0.0003*
Ferritin (mg/L)	715 (373-1249)	895 (397-1185)	739 (456-1384)	0.754
Troponin (ng/L)	13 (9-50)	14 (9-38)	10 (7-19)	0.131
D-Dimer (mg/L)	615 (283-1388)	595 (448-1016)	463 (280-905)	0.652
Procalcitonin (ng/ml)	0.3 (0.2-1.0)	0.5 (0.2-1.2)	0.3 (0.1-0.7)	0.402
HbA_1_C (mmol/mol)	50 (47- 58)	46 (43-51)	51 (41-70)	0.412
Lactate dehydrogenase (U/L)	1152 (847-1388)	985 (855-1225)	956 (812-1131)	0.213
Non-invasive respiratory support parameters				
Duration, hrs	9 (4-17)	43 (34-57)	132 (111-183)	<0.001*
Mode: CPAP only	17 (48.6%)	12 (34.3%)	12 (27.9%)	0.161
Mode: NIV only	11 (31.4%)	6 (17.1%)	4 (9.3%)	0.043*
Mode: Both	7 (20.0%)	17 (48.6%)	27 (62.8%)	0.001*
NIV: IPAP (cmH_2_O)	16 (12-20)	16 (14-20)	14 (13-16)	0.284
NIV: EPAP(cmH_2_O)	10 (10-12)	12 (10-12)	10 (10-12)	0.393
CPAP (cmH_2_O)	10 (8-12)	10 (10-12)	10 (8 - 12)	0.584

**Table 7:** Variable components of scoring systems APACHE II: Acute Physiology and Chronic Health Evaluation II score; GCS: Glasgow coma score; HACOR: heart rate, acidosis, consciousness, oxygenation, respiratory rate; IMV: invasive mechanical ventilation; MAP: mean arterial pressure; ROX: respiratory rate to oxygenation ratio; SOFA: sequential organ failure assessment.

Scoring system	Variable components
APACHE II	Temperature, MAP, heart rate, respiratory rate, oxygenation, arterial pH, GCS. Serum sodium, potassium, creatinine, haematocrit, white blood count. Age, history of severe organ insufficiency or immunocompromise.
SOFA score	PaO_2_/FiO_2_ ratio, IMV, platelet count, GCS, bilirubin, creatinine, MAP, or use of vasopressors
ROX index	(SpO_2_/FiO_2_) / respiratory rate
HACOR score	Heart rate, pH, GCS, PaO_2_/FiO_2_ ratio, respiratory rate
